# SEC23B Loss-of-Function Suppresses Hepcidin Expression by Impairing Glycosylation Pathway in Human Hepatic Cells

**DOI:** 10.3390/ijms23031304

**Published:** 2022-01-24

**Authors:** Barbara Eleni Rosato, Roberta Marra, Vanessa D’Onofrio, Federica Del Giudice, Simone Della Monica, Achille Iolascon, Immacolata Andolfo, Roberta Russo

**Affiliations:** 1 Dipartimento di Medicina Molecolare e Biotecnologie Mediche, Università degli Studi di Napoli Federico II, 80131 Napoli, Italy; rosato@ceinge.unina.it (B.E.R.); robertamarra.r@gmail.com (R.M.); vanessa.donofrio2@gmail.com (V.D.); simonedellamonica28@gmail.com (S.D.M.); achille.iolascon@unina.it (A.I.); 2 CEINGE Biotecnologie Avanzate, 80145 Napoli, Italy; delgiudicef@ceinge.unina.it

**Keywords:** congenital dyserythropoietic anemias, iron metabolism, *SEC23B*, glycosylation, hepcidin

## Abstract

Biallelic pathogenic variants in the *SEC23B* gene cause congenital dyserythropoietic anemia type II (CDA II), a rare hereditary disorder hallmarked by ineffective erythropoiesis, hemolysis, erythroblast morphological abnormalities, and hypo-glycosylation of some red blood cell membrane proteins. Abnormalities in *SEC23B*, which encodes the homonymous cytoplasmic COPII (coat protein complex II) component, disturb the endoplasmic reticulum to Golgi trafficking and affect different glycosylation pathways. The most harmful complication of CDA II is the severe iron overload. Within our case series (28 CDA II patients), approximately 36% of them exhibit severe iron overload despite mild degree of anemia and slightly increased levels of ERFE (the only erythroid regulator of hepcidin suppression). Thus, we hypothesized a direct role of SEC23B loss-of-function in the pathomechanism of hepatic iron overload. We established a hepatic cell line, HuH7, stably silenced for *SEC23B*. In silenced cells, we observed significant alterations of the iron status, due to both the alteration in BMP/SMADs pathway effectors and a reduced capability to sense BMP6 stimulus. We demonstrated that the loss-of-function of SEC23B is responsible of the impairment in glycosylation of the membrane proteins involved in the activation of the BMP/SMADs pathway with subsequent hepcidin suppression. Most of these data were confirmed in another hepatic cell line, HepG2, stably silenced for *SEC23B*. Our findings suggested that the pathogenic mechanism of iron overload in CDA II is associated to both ineffective erythropoiesis and to a specific involvement of *SEC23B* pathogenic variants at hepatic level. Finally, we demonstrated the ability of *SEC23B* paralog, i.e., *SEC23A*, to rescue the hepcidin suppression, highlighting the functional overlap between the two SEC23 paralogs in human hepatic cells.

## 1. Introduction

Congenital dyserythropoietic anemias (CDAs) are a group of rare hereditary disorders belonging to a subtype of bone marrow failure syndromes, characterized by monolineage involvement and morphological abnormalities in erythroid precursor cells [1]. CDAs are defined by maturation arrest of the erythroid lineage with the consequent reduced production of erythrocytes [1,2]. Among them, congenital dyserythropoietic anemia type II (CDA II) is the most common form. CDA II is an autosomal recessive disease characterized by iron-loading-anemia with relative reticulocytopenia (ineffective erythropoiesis) [3]. It is also characterized by the presence of bi- and multinucleated erythroblasts in bone marrow, with nuclei of equal size and DNA content [4]. Analysis of red cell membrane proteins by sodium dodecyl sulfate polyacrylamide gel electrophoresis (SDS-PAGE) identifies glycosylation abnormalities with fast moving band 3 (anion exchanger 1) and band 4.5 (glucose transporter 1) [5,6]. This is due to the main biochemical feature of this hereditary disease: defective glycosylation [7]. Structural data of erythrocyte N-glycans indicate that CDA II is not a distinct glycosylation disorder but is caused by a defect disturbing Golgi processing in erythroblasts [8]. CDA II results from homozygous or compound heterozygous loss-of-function mutations in *SEC23B* [9,10,11], one of the two paralogous *SEC23* genes (*SEC23A* and *SEC23B*) that encodes a core component of COPII (coat protein complex II) vesicles [7]. COPII is a multi-subunit complex that mediates accumulation of secretory cargo, deformation of the membrane, and generation of subsequent anterograde transport of correctly folded cargo that bud from the ER towards the Golgi apparatus [12]. This pathway is critical for membrane homeostasis, localization of proteins within cells, and secretion of extracellular factors. Therapeutic strategies for CDA II only include supportive therapies, such as transfusion, splenectomy, and hematopoietic stem-cell transplantation in transfusion-dependent patients [1,13,14].

The worst complication for patients with CDA II is the iron overload [15]. It has been reported that ineffective erythropoiesis results in strong down-regulation of the hepatic hormone hepcidin, increased iron absorption, and systemic iron overload, which is mediated by the erythroid hormone erythroferrone (ERFE) [16,17,18,19]. ERFE expression in CDA II patients is increased and related to abnormal erythropoiesis and acts by inhibiting hepatic signaling of BMP/SMADs [20,21]. The activation of the BMP/SMADs signaling requires, at hepatic level, the assembly of a multi-protein complex that senses and regulates the intracellular pathway that induces hepcidin transcription [22,23]. Although over-expression of ERFE was well documented in CDA II patients, no clear correlation between ERFE levels and iron balance has been proven so far [18,24]. This may suggest that ERFE cannot be the only regulator of hepcidin suppression in these patients.

We herein described a subset of CDA II patients showing iron overload even in presence of a mild degree of anemia and slightly increased levels of ERFE. Thus, we hypothesized a specific role of SEC23B loss-of-function in impairing iron metabolism at hepatic level. We demonstrate that the loss-of-function of SEC23B impairs the glycosylation of membrane proteins involved in the activation of the BMP/SMADs signaling with subsequent hepcidin suppression in HuH7 cell line, a human hepatic model, stably silenced for *SEC23B* gene. Most of these data were confirmed in another hepatic cell line, HepG2, stably silenced for *SEC23B*.

## 2. Results

### 2.1. Analysis of Iron Balance in Patients with CDA II

**Figure 1:**
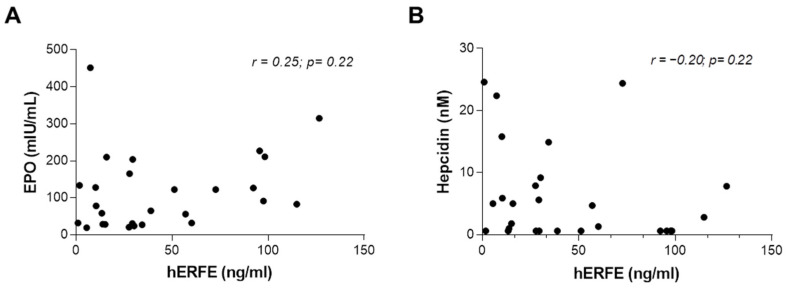
**Correlation analysis between ERFE, EPO, and hepcidin levels.** The graphs show the correlation analysis between ERFE and EPO levels (**A**) and between hepcidin and ERFE levels (**B**) in plasma samples from 28 CDA II enrolled in the study (r and *p*-value by Pearson correlation analysis).

Within our cohort of patients with CDA II enrolled in our Registry of hereditary anemias [15,25,26], we selected 28 cases showing iron overload, defined as transferrin saturation (TSAT) > 45%. We further stratified these patients into two subgroups according to the degree of anemia: (i) mild (Hb ≥ 10.0 g/dL) and (ii) moderate/severe (Hb < 10.0 g/dL) (Table 1). As expected, overall patients exhibited reduced hepcidin levels and high ferritin levels compared to reference ranges (Table 1). Moreover, all patients showed augmented EPO levels compared to reference range with a statistically significant increase in moderate/severe anemic subjects compared to the mild anemic ones. Nevertheless, we did not observe any difference in the ERFE levels between the two subgroups (Table 1). To further investigate on ERFE levels, we performed a correlation analysis on the entire case series between ERFE and EPO levels. Our data showed a slight, non-statistically significant, correlation between EPO and ERFE levels (Figure 1A). Similarly, we observed a negative correlation between hepcidin plasma levels and ERFE ones although the analysis did not reach the statistical significance (Figure 1B).

### 2.2. Assessment of Iron-Related Genes in HuH7 Cells Stably Silenced for SEC23B

**Figure 2:**
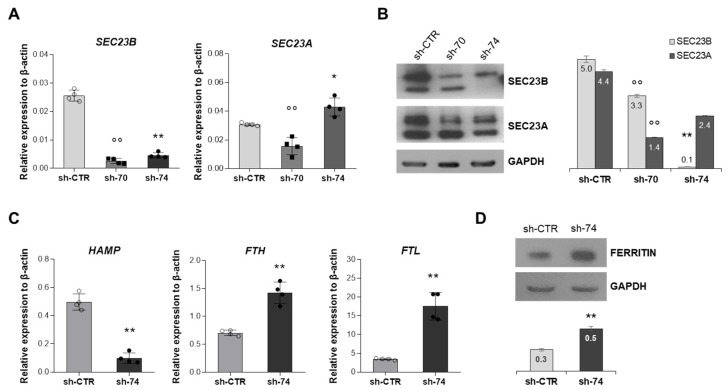
**Characterization of HuH7 silenced for *SEC23B*.** (**A**) *SEC23B* and *SEC23A* gene expression in HuH7 clones silenced for *SEC23B*, sh-*SEC23B*-70 (sh-70), and sh-*SEC23B*-74 (sh-74) compared to control cells (sh-CTR). Data are means ± standard deviation of three independent experiments (* *p* < 0.05, sh-CTR vs. sh-74; ** *p* < 0.01, sh-CTR vs. sh-74; °° *p* < 0.01, sh-CTR vs. sh-70; Student’s *t*-test). (**B**) Representatives immunoblot of SEC23B and SEC23A in HuH7 sh-70, sh-74, and sh-CTR cells (left panel) and densitometry quantification (right panel). Data are means ± standard deviation of three independent experiments (** *p* < 0.01, sh-CTR vs. sh-74; °° *p* < 0.01, sh-CTR vs. sh-70; Student’s *t*-test). (**C**) *HAMP*, *FTL*, and *FTH* gene expression in HuH7 sh-74 compared to HuH7 sh-CTR cells. Data are means ± standard deviation of three independent experiments (** *p* < 0.01; Student’s *t*-test). (**D**) Representatives immunoblot of ferritin in HuH7 sh-74 compared to HuH7 sh-CTR cells and densitometry quantification. Data are means ± standard deviation of three independent experiments (** *p* < 0.01; Student’s *t*-test).

ERFE levels were inadequate to explain hepcidin suppression in CDA II patients, so we investigated a possible specific role of SEC23B in hepcidin regulation. To reproduce *SEC23B* loss-of-function at hepatic level, HuH7 cell lines stably silenced for *SEC23B* were generated by the means of small hairpin RNA (shRNA) method. The silencing efficiency was tested by qRT-PCR and Western blot analyses on both SEC23B and its paralogue SEC23A (Figure 2A,B). We observed downregulation of *SEC23B* expression in both sh-*SEC23B*-74 and sh-*SEC23B*-70 clones compared to sh-CTR clone. Of note, the two silenced clones for *SEC23B* showed different behaviors in terms of expression of *SEC23A*. Indeed, there was significant downregulation of *SEC23A* in the HuH7 sh-*SEC23B*-70 cells, which suggested a less-specific effect of the silencing in the HuH7 sh-*SEC23B*-70 cells (Figure 2A,B), whereas we observed a marked up-regulation of *SEC23A* in the HuH7 sh-*SEC23B*-74 cells compared to Sh-CTR ones, as expected for a CDA II model. Indeed, as observed in some CDA II patients, low *SEC23B* expression alleles result in a compensatory increase in the paralog gene, *SEC23A* [28].

The silencing experiment was carried out also in another human hepatic cell line, i.e., HepG2 cells. Of note, we observed comparable results using both shRNAs. The sh-*SEC23B*-74 clone showed higher efficiency and specificity for *SEC23B* silencing compared to the sh-*SEC23B*-70 also in HepG2 cells (Figure S1A,B). For this reason, we chose this sh-*SEC23B*-74 for further analysis.

In the HuH7 sh-*SEC23B*-74 clone, we found reduced expression of *HAMP* gene and increased expression of *FTH* and *FTL* genes compared to sh-CTR cells (Figure 2C,D). Protein expression analysis also showed an increase of ferritin expression in silenced cells compared to control ones (Figure 2D). Similarly, we observed reduced *HAMP* gene expression and increased expression of ferritin in HepG2 sh-*SEC23B*-74 cells compared to sh-CTR cells (Figure S1C,D).

### 2.3. SEC23B Loss-of-Function Alters BMP/SMADs Pathway and Fails to Activate the Signaling in Response to BMP6

**Figure 3:**
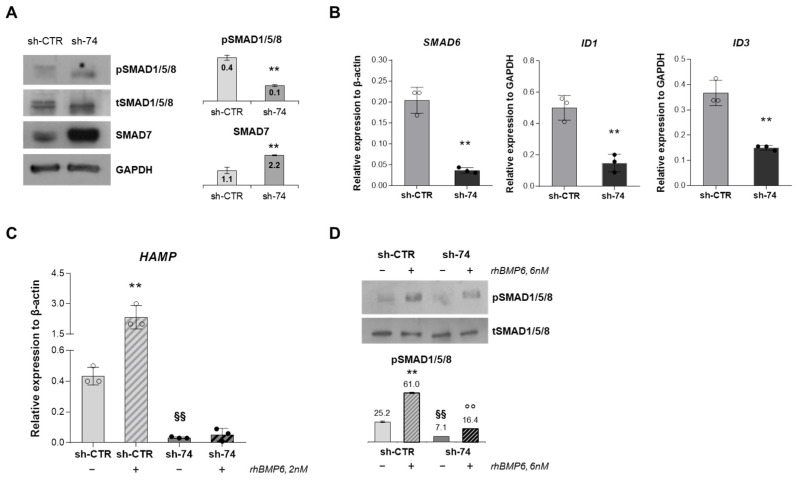
**Evaluation of the activation of BMP/SMADs pathway in SEC23B-silenced HuH7**. (**A**) **Left panel**. Representative immunoblots of pSMAD1/5/8, tSMAD1/5/8 and SMAD7 proteins in total cells lysate of HuH7 sh-74 and sh-CTR cells. GAPDH is the loading control. **Right panel**. Quantification by densitometric analysis of three separate Western blots with similar results. Data are means ± standard deviation (** *p* < 0.01; Student’s *t*-test). (**B**) *SMAD6*, *ID1*, and *ID3* gene expression in HuH7 sh-74 and sh-CTR cells. Data are means ± standard deviation of three independent experiments. (** *p* < 0.01; Student’s *t*-test). (**C**) *HAMP* gene expression in control HuH7 and sh74-*SEC23B* HuH7 cells treated or not with rhBMP6 (2 nM). Data are means ± standard deviation of three independent experiments. (** *p* < 0.01, sh-CTR + vehicle vs. sh-CTR + rhBMP6 (2 nM); ANOVA test and post-hoc correction by Tukey’s multiple comparison tests; ^§§^ *p* < 0.01, sh-CTR vs. sh-74; Student’s *t*-test). (**D**) Representative immunoblots of pSMAD1/5/8 and tSMAD1/5/8 proteins in total lysate of HuH7 sh-74 and sh-CTR cells treated (+) or not (−) with rhBMP6 (6nM). Densitometric analysis of three separate Western blots with similar results is shown (** *p* < 0.01, sh-CTR + vehicle vs. sh-CTR + rhBMP6 (6 nM); °° *p* < 0.01, sh-CTR + vehicle vs. sh-CTR + rhBMP6 (6 nM); ^§§^ *p* < 0.01, sh-CTR vs. sh-74; Student’s *t*-test).

To further characterize the alteration of iron status in *SEC23B*-silenced HuH7 cells, we evaluated the main components of the BMP/SMADs pathway, which is the main regulatory pathway of *HAMP* gene expression [23] Firstly, we evaluated the phosphorylation levels of SMAD1/5/8 (pSMAD1/5/8, the receptor-activated effector of the pathway) and the protein expression of SMAD7 (inhibitor of the signaling) in our cellular system. We found strongly reduced levels of pSMAD1/5/8 and increased expression of SMAD7 in HuH7 sh-*SEC23B*-74 cells compared to control ones (Figure 3A). Similar results were obtained in HepG2 sh-*SEC23B*-74 cells compared to control ones (Figure S2A).

The inhibition of BMP/SMADs pathway was also assessed by gene expression analysis of some target genes. Particularly, *SMAD6*, *ID1,* and *ID3* gene expression resulted down-regulated in HuH7 sh-*SEC23B*-74 cells compared to control cells (Figure 3B).

Finally, we tested whether the loss of SEC23B affected the BMP/SMADs pathway activation. To this aim, we treated both sh-CTR and sh-*SEC23B*-74 clones with recombinant BMP6. Liver endothelial cells regulate *HAMP* transcription by producing BMP6, which activates the intracellular BMP/SMADs signaling [29]. In BMP6 treated cells, we evaluated both the *HAMP* gene expression and pSMAD1/5/8 levels. As expected, we observed a strong increase in *HAMP* expression and pSMAD1/5/8 levels in HuH7 sh-CTR cells treated with BMP6 compared to non-treated ones. Conversely, the BMP6 treatment did not increase *HAMP* gene expression in HuH7 sh-*SEC23B*-74 cells treated with BMP6 compared to sh-*SEC23B*-74 not-treated cells (Figure 3C). Moreover, we observed only a slight increase of phosphorylation levels of SMAD1/5/8 in HuH7 sh-*SEC23B*-74 cells treated with BMP6 compared to sh-*SEC23B*-74 not-treated cells (Figure 3D). These data suggested that *SEC23B*-silenced cells were not responsive to BMP6 stimulus.

### 2.4. Inhibition of N-Glycosylation Accounts for Reduced Activation of BMP/SMADs Pathway

**Figure 4:**
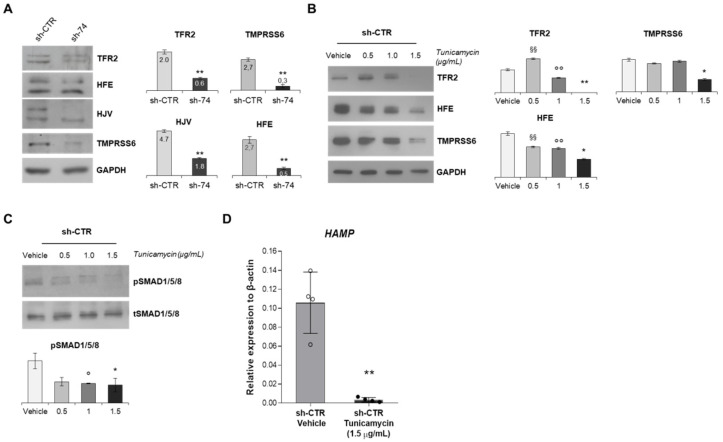
**N-linked glycosylation is required for proper BMP/SMADs signaling**. (**A**) Left panel. Representative immunoblots of TFR2, HFE, HJV, and TMPRSS6 proteins in total cells lysate of HuH7 sh-74 and sh-CTR cells. GAPDH is the loading control. Right panel. Quantification by densitometric analysis of three separate Western blots with similar results. Data are means ± standard deviation. (** *p* < 0.01; Student’s *t*-test). (**B**) **Left panel**. Representative immunoblots of TFR2, HFE, and TMPRSS6 in HuH7 control cells treated with DMSO (vehicle) or increasing concentration of tunicamycin (0.5, 1.0, 1.5 µg/mL). GAPDH is the loading control. **Right panel**. Quantification by densitometric analysis of three separate Western blots with similar results. Data are means ± standard deviation. (** *p* < 0.01, 1.5 μg/mL tunicamycin-treated cells vs. vehicle, °° *p* < 0.01 of 1.0 µg/mL tunicamycin-treated cells vs. vehicle, ^§§^ *p* < 0.01 0.5 ug/mL tunicamycin-treated cells vs. vehicle; * *p* < 0.05, 1.5 μg/mL tunicamycin-treated cells vs. vehicle; ANOVA test and post-hoc correction by Tukey’s multiple comparison tests). (**C**) **Upper panel**. Representative immunoblots of pSMAD1/5/8 and tSMAD1/5/8 proteins in HuH7 control cells treated with DMSO (vehicle) and increasing concentration of tunicamycin (0.5, 1.0, 1.5 µg/mL). **Lower panel**. Quantification by densitometric analysis of three separate Western blots with similar results. Data are means ± standard deviation (* *p* < 0.05, 1.5 μg/mL tunicamycin-treated cells vs. vehicle, ° *p* < 0.05 of 1.0 µg/mL tunicamycin-treated cells vs. vehicle. ANOVA test and post-hoc correction by Tukey’s multiple comparison tests). (**D**) Quantification of *HAMP* gene expression in sh-CTR cells treated with DMSO and tunicamycin (1.5 µg/mL). Data are means ± standard deviation of three independent experiments. (** *p* < 0.01; Student’s *t*-test).

To understand the absence of activation of BMP/SMADs pathway after BMP6 stimulation in *SEC23B*-silenced cells, we further investigated the signaling pathway by assessing the protein expression of the main components of the multiprotein membrane complex (composed by TFR2, HFE, and HJV) that transduces the external BMP6 stimulus inside cells. We demonstrated a decreased protein expression of TFR2, HFE, and HJV in HuH7 sh-*SEC23B*-74 cells compared to control cells by Western blotting (WB) analysis (Figure 4A). Comparable results were obtained in HepG2 sh-*SEC23B*-74 cells compared to control ones (Figure S2B). Interestingly, a decreased protein expression of the negative regulator of the BMP/SMADs signaling, i.e., TMPRSS6, was also observed in both HuH7- and HepG2-silenced cells (Figure 4A and Figure S2B).

Abnormalities in SEC23B affect different glycosylation pathways in CDA II. We hypothesized a role of impaired glycosylation of the multiprotein membrane complex proteins as causative of their altered expression. As proof of concept, we treated HuH7 control cells with increasing concentrations of tunicamycin (0.5, 1.0, 1.5 µg/mL), a drug that blocks N-linked glycosylation. Of note, N-linked glycosylation is necessary for the correct folding of the multiprotein membrane complex. We found a direct correlation between the increased concentration of tunicamycin and the progressive reduction of protein expression of TFR2, TMPRSS6, and HFE (Figure 4B). As consequence of the downregulation of this membrane complex, we observed a marked reduction of the phosphorylation levels of SMAD1/5/8 in the cells treated with tunicamycin (Figure 4C). The final effect of this treatment was the downregulation of *HAMP* gene expression (Figure 4D).

### 2.5. SEC23A Over-Expression Rescues the Impaired BMP/SMADs Signaling

**Figure 5:**
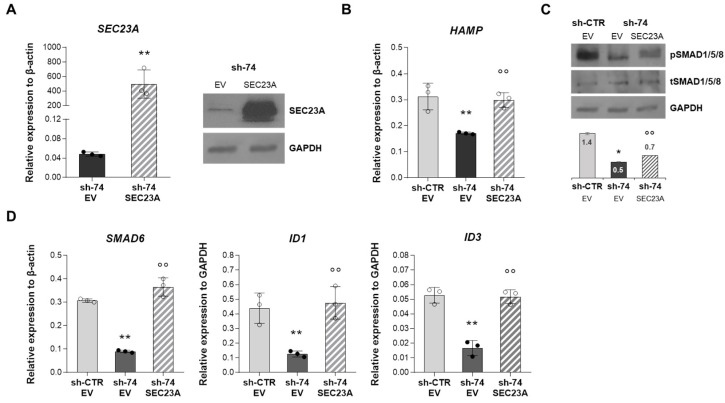
**SEC23A overexpression in HuH7 *SEC23B*-silenced cells**. (**A**) **Left panel**. *SEC23A* gene expression in sh-74 cells over-expressing empty vector (EV) and *SEC23A* coding sequence. Data are means ± standard deviation of three independent experiments. (** *p* < 0.01, sh-74 + EV vs. sh-74 + SEC23A; Student’s *t*-test). **Right panel**. Representatives immunoblot of SEC23A protein in total cells lysate of sh-74 HuH7 cells over-expressing EV and SEC23A. (**B**) *HAMP* gene expression in sh-CTR HuH7 cells treated with EV, compared to both sh-74 + EV and sh-74 + SEC23A HuH7 cells. Data are means ± standard deviation of three independent experiments. (** *p* > 0.01, sh-CTR + EV vs. sh-74 + EV, °° *p* < 0.01, sh-74 + EV vs. sh-74 + SEC23A; ANOVA test and post-hoc correction by Tukey’s multiple comparison tests). (**C**) Representatives immunoblot of pSMAD1/5/8 and tSMAD1/5/8 proteins in total cell lysate of sh-CTR HuH7 cells treated with EV, compared to both sh-74 + EV and sh-74 + SEC23A HuH7 cells. GAPDH is used as loading control (* *p* < 0.05, sh-CTR + EV vs. sh-74 + EV, °° *p* < 0.01, sh-74 + EV vs. sh-74 + SEC23A; ANOVA test and post-hoc correction by Tukey’s multiple comparison tests). (**D**) *SMAD6, ID1,* and *ID3* gene expression in sh-CTR HuH7 cells treated with EV, compared to both sh-74 + EV and sh-74 + SEC23A HuH7 cells. Data are means ± standard deviation of three independent experiments (** *p* > 0.01, sh-CTR + EV vs. sh-74 + EV, °° *p* < 0.01, sh-74 + EV vs. sh-74 + SEC23A; ANOVA test and post-hoc correction by Tukey’s multiple comparison tests).

We performed a rescue experiment by overexpressing the SEC23B paralog, SEC23A, in HuH7 *SEC23B*-silenced cells. Indeed, it was demonstrated that low *SEC23B* expression results in a compensatory increase in the paralog gene, *SEC23A* in CDA II patients [28]. To this aim, human *SEC23A* full-length ORF was transfected into HuH7 sh-*SEC23B*-74 cells for 48 h. The over-expression of SEC23A was verified at both mRNA and protein level (Figure 5A). Interestingly, the overexpression of SEC23A rescued the downregulation of *HAMP* gene expression in *SEC23B*-silenced cells (Figure 5B). Additionally, we observed increased phosphorylation levels of SMAD1/5/8 (Figure 5C) with subsequent increased expression of *SMAD6*, *ID1,* and *ID3* genes in *SEC23B*-silenced cells transiently transfected with *SEC23A* (Figure 5D).

## 3. Discussion

Congenital dyserythropoietic anemia type II is the most frequent subtype among the CDAs, a heterogeneous group of rare hereditary anemias hallmarked by ineffective erythropoiesis and iron overload. Most CDA II patients are mildly affected. However, despite the monogenic origin of the disease, a high clinical heterogeneity among affected subjects was observed. This high clinical heterogeneity can be partially explained with co-inheritance of additional genetic variants in modifier genes [3,10,11,15]. Indeed, approximately 30% of CDA II patients exhibit a tendency to iron overload (ferritin > 300 ng/mL), while 17% of them show marked hemosiderosis (ferritin > 600 ng/mL) even regardless of the regimen of transfusion dependence [15].

The pathogenetic mechanism of iron overload was associated to expanding abnormal erythropoiesis that stimulates iron absorption in CDA II [15,21,24,30]. It is now clear that stimulated erythrocyte precursors produce one or more hepcidin-suppressive factors [31,32]. Currently, the main known erythroid regulator involved in hepcidin regulation is ERFE [16,17,18,19,21,33,34]. Erythroferrone mediates the suppression of *HAMP* expression by acting as ligand trap for BMPs, blocking the activation of the BMP/SMADs pathway [18,23,33,35]. In our previous studies, we demonstrated the overexpression of ERFE as an important determinant of altered iron balance in CDA II patients [18,19,36]. Nevertheless, we did not observe a clear correlation between ERFE levels and CDA II iron balance, thus suggesting that ERFE cannot be the only erythroid regulator of hepcidin suppression in these patients [19]. We herein described a case series of 28 CDA II patients showing iron overload with high transferrin saturation and very low levels of hepcidin. Of note, 36% (10/28) of them showed mild degree of anemia and slightly increased levels of ERFE despite the high EPO levels. Accordingly, we did not observe any correlation between ERFE and EPO levels in this case series, suggesting that additional elements take part in hepatic iron overload in CDA II.

Since *SEC23B* is a ubiquitously expressed gene, we hypothesized a direct role of its loss-of-function in hepcidin regulation at hepatic level. We firstly established two hepatic cell line, HuH7 and HepG2, stably silenced for *SEC23B*. We demonstrated that silencing of *SEC23B* leads to impaired activation of BMP/SMADs signaling pathway, which is responsible of hepcidin transcription [20,21,22,23,37,38]. Indeed, in silenced cells, we observed a reduction of the phosphorylation levels of SMAD1/5/8 that leads to the downregulation of the BMP/SMADs target genes, i.e., *HAMP*, *SMAD6*, *ID1,* and *ID3*. Moreover, we also observed an increased expression of ferritin, which correlates with the high levels of ferritinemia encountered in the patients with CDA II.

**Figure 6:**
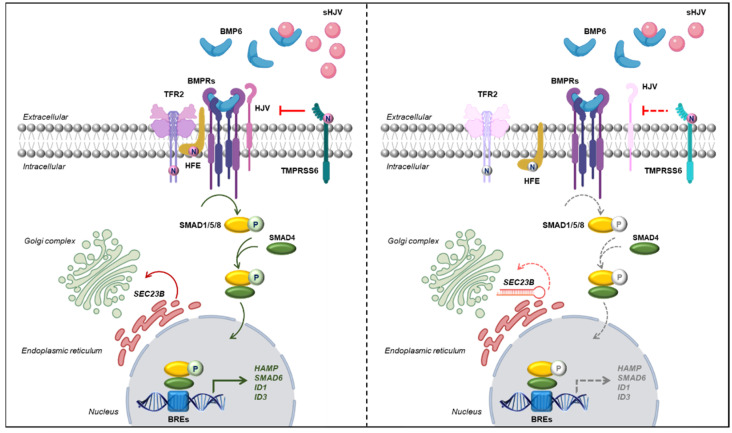
**Schematic models of the mechanism of iron overload in *SEC23B* loss-of-function hepatic cells**. **Left panel**. At physiological conditions, SEC23B mediates the anterograde trafficking of correctly folded proteins and post-translational modification. Correctly processed proteins (TFR2, HJV, HFE) associated in the membrane complex that mediates activation of BMP/SMADs pathway. The activation of the complex results in the phosphorylation of SMAD1/5/8 that in turn mediates the activation of the transcription of the BMP target genes, *HAMP*, *SMAD6*, *ID1*, and *ID3*. **Right panel**. *SEC23B* loss-of-function determines the decreased glycosylation of membrane proteins TFR2, HFE, and HJV (light colored), which accounts for the reduced expression and stabilization of the membrane complex. Therefore, BMP6 failed to activate the pathway, and this finally causes the suppression of *HAMP* gene transcription.

The suppression of hepcidin by ERFE is mediated by interference with paracrine BMP/SMADs signaling that regulates hepcidin transcription in hepatocytes [17]. To mimic this physiological mechanism, we tested the efficiency of HuH7 sh-*SEC23B*-74 cells in activating the pathway after BMP6 stimulation. We observed that HuH7 *SEC23B*-silenced cells did not activate the signaling after BMP6 stimulus compared to control cells. These data suggested that *SEC23B*-silenced cells failed to transduce external BMP6 stimulus inside the cytoplasm. The link between the extracellular BMP6-dependent signal and the activation of pSMAD1/5/8 requires the assembly of a multiprotein complex that regulates the pathway [20]. The hereditary hemochromatosis-associated membrane proteins HFE, TFR2, and HJV are required for adequate hepatic expression of the iron hormone hepcidin [39,40,41]. Moreover, TMPRSS6 acts as negative regulator of the pathway by the cleavage of HJV [42,43]. In our *SEC23B*-silenced cells, HFE, TFR2, TMPRSS6, and HJV were strongly down-regulated compared to control cells. These proteins have in common the complex post-translational modification that requires N-glycosylation for the correct localization (HFE), activation (TMPRSS6), and stabilization (TFR2) in the plasma membrane [44,45,46]. As proof of concept, we treated HuH7 cells with tunicamycin, an N-linked glycosylation inhibitor [47]. Treatment with increasing concentrations of tunicamycin demonstrated that the altered glycosylation led to a reduced expression of TFR2, HFE, and TMPRSS6 proteins. Furthermore, also SMAD1/5/8 phosphorylation was strongly reduced after tunicamycin treatment, leading to suppressed *HAMP* gene expression. These data suggest that altered glycosylation is directly involved in regulation of hepatic iron metabolism (Figure 6). Our data are in agreement with the biochemical hallmark of CDA II, the hypoglycosylation of band 3 on red blood cell surface, which suggests defective glycosylation as pathogenetic mechanism of this disorder. Additionally, this defective glycosylation is not confined to erythrocyte compartment since altered glycosylation was also observed for TFR1 and TFR2 proteins [46].

This study also highlighted for the first time the functional overlap between the two SEC23 paralogs in human hepatic cells. Indeed, we demonstrated that overexpression of *SEC23A* in *SEC23B*-silenced cells completely rescued the observed phenotype by re-activating BMP/SMADs signaling pathway and *HAMP* transcription. This is in agreement with a recent study on an erythroid SEC23B-deficient cellular model (HUDEP-2), whose CDA II phenotype was rescued by increased expression of SEC23A [48]. These results are highly relevant to suggest novel therapeutic approach for CDA II. Indeed, in-vitro gene therapy with lentiviral transduction of p60-BBF2H7, a transactivator of Sec23a, has been shown to compensate for mutated *SEC23B* in primary human erythroblasts isolated from CDA II patients [49].

Our data strongly support the concept that increasing the expression of the SEC23A gene may prove a novel therapeutic strategy for CDA II not only for the treatment of anemia but also of the iron overload that remains the most harmful complication of this condition.

## 4. Materials and Methods

### 4.1. Patients

Twenty-eight patients with CDA II were enrolled in the study, all showing iron overload defined as TSAT > 45%. CDA II diagnosis was based on clinical findings and biochemical and molecular analyses, as previously reported [2,3,4,24,25]. Patients were furtherly stratified into two age- and gender-matched subgroups based on Hb levels: (i) mildly affected subjects (Hb ≥ 10.0 g/dL; *n* = 10) and (ii) moderately/severely affected cases (Hb < 10.0 g/dL; *n* = 18). Overall clinical features of enrolled patients are shown in Table 1. The Ethical Committee of University of Naples approved the collection of the patient data from the Medical Genetics Ambulatory in Naples (DAIMedLab, ‘Federico II’ University, protocol n.252/18 October 2018). Plasma samples from the patients were obtained after signed informed consent and according to the Declaration of Helsinki.

### 4.2. Hepcidin, ERFE, and Erythropoietin Levels in Plasma Samples

Plasma samples were collected from peripheral blood of patients and healthy controls after signed informed consent, according to Declaration of Helsinki. Plasma levels of Hepcidin (Intrinsic HEPCIDIN IDx, Intrinsic Lifesciences), human ERFE (hERFE) (Intrinsic Erythroferrone IE; Intrinsic Lifesciences, La Jolla, California, USA), erythropoietin (EPO) (Quantikine IVD ELISA Human Erythropoietin), and sTfR (Quantikine IVD ELISA Human sTfR, R&D System Minneapolis, Minnesota, USA) were quantified using ELISA kits. The concentration of each parameter in each sample was determined through the fitting of a four-parameter logistic curve, according to the manufacturer protocols.

### 4.3. Production of Lentiviral Particles and Infection of the HuH7 and HepG2 Cell Line

Knock-down of SEC23B expression was obtained through infection of lentiviral particles that targeted human *SEC23B* of HuH7 and HepG2 hepatic carcinoma cell lines, as previously described [50]. After 48 h, Puromycin (2 μg/mL) was added as a selection marker. Single, resistant colonies were transferred in a 96-well and then expanded. The infection efficiency was assessed by testing gene and protein expression of SEC23B and SEC23A compared to control HUH7/HepG2 cells.

### 4.4. Culture of Cell Lines and Drugs Treatments

HuH7 (differentiated human hepatoma) cell line was obtained from Japan Health Science Research Resources Bank (Rinku Town, Osaka, Japan) and previously used [18,36]. The wild-type (control) HuH7 cells and SEC23B-silenced HuH7 stable clones were maintained in Dulbecco’s modified Eagle’s medium (Sigma Aldrich, Milan, Italy), supplemented with 10% (*v*/*v*) fetal bovine serum (Life Technologies; CA, USA), 100 U/mL penicillin (Life Technologies, Carlsbad, California, USA), and 100 mg/mL streptomycin (Life Technologies) at 37 °C in humidified air/CO2 (19:1) atmosphere. HepG2 (hepatocyte carcinoma) cells were obtained from American Type Culture Collection (ATCC, Manassas, VA, United States) and maintained following the manufacturer’s instruction. [18,51]. The wild-type (control) HepG2 cells and *SEC23B*-silenced HepG2 stable clones were cultured in RPMI 1640 (Sigma Aldrich, Milan, Italy) supplemented with 10% (*v*/*v*) fetal bovine serum (Life Technologies), 100 U/mL penicillin (Life Technologies), and 100 mg/mL streptomycin (Life Technologies), at 37°C in humidified air/CO2 (19:1) atmosphere.

Recombinant human BMP6 protein (507-BP-020; R&D Systems, Minneapolis, MN, USA) was used at 2 nM (90 min) and 6 nM (30 min) [33]. Tunicamycin (T7765, Sigma Aldrich) was added at increasing concentration (0.5, 1.0, 1.5 µg/mL) [52]; DMSO was used as a vehicle. Precision LentiORF Human SEC23A (3 µg) (Horizon) was transiently transfected into SEC23B-silenced HuH7 using FuGENE HD transfection Reagent (Promega, Cells were harvested at 0, 24, and 48 h.

### 4.5. Gene Expression Analysis

Total RNA was extracted from cells using TRIzol reagent (Life Technologies, Waltham, MA, USA). Synthesis of cDNA from total RNA (1 μg) was performed using cDNA synthesis kits (Life Technologies). Quantitative real-time PCR (qRT-PCR) analysis was carried out using Power SYBR Green PCR Master Mix (Life Technologies) to evaluate the expression of *SEC23B*, *SEC23A*, *FTH*, *FTL*, *HAMP*, and *SMAD6* genes. β-*Actin* and *GAPDH* genes were used as the internal controls. The primers were designed with the Primer Express 2.1 software (Applied Biosystems, Waltham, Massachusetts, Stati Unit). Primer sequences are available upon request (roberta.russo@unina.it). Expression analysis of *ID1* and *ID3* genes was performed using a standard TaqMan PCR kit protocol. (*ID1*: Hs03676575_s1; *ID3*: Hs00171409_m1; GAPDH Hs99999905_m1; Applied Biosystem) The samples were amplified by 7900HT Sequence Detection System (Applied Biosystems) using standard cycling conditions. Relative gene expression was calculated either by the 2^-ΔCt^ method, with the fold change determined using the ratio between the gene expression and the internal control, or by the mean fold change 2^-(average ΔΔCt)^, determined using the mean difference in ΔCt between the gene and the internal control.

### 4.6. Protein Expression Analysis

Proteins were extracted from the cells using RIPA lysis buffer in the presence of a protease inhibitor cocktail (Roche, Rotkreuz, Switzerland). Protein extracts have been quantified using Bradford dye-binding method (BioRad, Milan, Italy). Total protein extracts were analyzed by SDS-PAGE, transferred to polyvinylidene difluoride membranes (BioRad, Milan, Italy), and then incubated with the required combinations of the following antibodies: rabbit anti-SEC23B (1:1000; SAB2102104; Sigma Aldrich); SEC23A; Rabbit anti-Ferritin (1:1000; Abcam, ab75973); polyclonal rabbit Anti-Transferrin Receptor 2 (1:1000, Abcam, ab80194); monoclonal rabbit anti-HFE (1:1000. Abcam, ab133639); monoclonal rabbit anti-TMPRSS6 (1:1000, Abcam, ab56180); monoclonal rabbit anti-pSMAD 1/5/8 (1:1000, Cell Signalling, 13820); polyclonal rabbit anti-tSMAD 1/5/8/9 (1:1000, abcam, ab13723); rabbit anti-GAPDH (1:1000; 2118, Cell Signaling Technology Danvers, Massachusetts, USA); and Rabbit anti-HJV, which was kindly gifted by Laura Silvestri. Semi-quantitative analysis of protein expression was performed as previously described [36]. The bands were quantified using the Quantity One software (BioRad, Milan, Italy) to obtain integrated optical densities, which were then normalized to GAPDH.

### 4.7. Statistical Analysis

Statistical significances of the differences in protein and gene expression were determined using Student’s *t*-tests. Statistical significances of multiple comparisons were calculated using ANOVA, and post-hoc correction was performed using Tukey’s multiple comparison tests. A two-sided *p* < 0.05 was considered statistically significant.

## 5. Conclusions

We herein demonstrated, for the first time, a direct role of *SEC23B* loss-of-function variants as contributing cause of CDA II-related iron overload at hepatic level. Our data might explain CDA II cases showing marked iron overload despite mild or slight degree of anemia where the role of ERFE seems to be secondary. Moreover, understanding the molecular mechanism that underlies *SEC23B* loss-of-function at hepatic level shed light on new therapeutic strategies of iron overload in CDA II.

## Figures and Tables

**Table 1:** CDA II patients enrolled in the study.

Analysis	Units	Chronic Anemia		*p*-Value ^†^	Reference
		Mild	Moderate/Severe		Range
		Hb ≥ 10.0 g/dL	Hb < 10.0 g/dL		
N		10	18	-	-
Gender	male/female	3 (0.3)/7 (0.7)	10 (0.6)/8 (0.4)	0.19	-
Age at sampling	years	28.6 ± 4.3	17.0 ± 5.7	0.19	-
Hemoglobin	g/dL	10.9 ± 0.2	8.6 ± 0.3	0.00001	11.5–15.5
ARC	×10^3^/µL	51.5 ± 6.0	77.7 ± 14.7	0.21	20–90
TSAT	%	90.5 ± 6.5	76.7 ± 4.6	0.09	15–39
hERFE	ng/mL	40.5 ± 11.9	43.2 ± 9.3	0.86	0.1–3.8
EPO	mIU/mL	51.6 ± 11.1	151.1 ± 27.4	0.01	3.1–14.9
sTfR	mg/L	3.8 ± 0.5	4.2 ± 0.5	0.59	0.78–1.89
Hepcidin	nM	5.6 ± 2.3	6.2 ± 1.9	0.85	male: 40.10
					female: 23.27
Hepcidin/ferritin	-	0.02 ± 0.01	0.03 ± 0.01	0.46	-
Ferritin	ng/mL	559.7 ± 234.4	369.2 ± 115.2	0.42	22.0–275.0
Ferritin/age ^§^	-	20.5 ± 6.9	57.9 ± 19.9	0.21	-

ARC, absolute reticulocyte count; TSAT, transferrin saturation; sTfR, soluble transferrin saturation; EPO, erythropoietin; hERFE, human erythroferrone. Quantitative variables data are presented as mean ± SEM. Qualitative variables data are presented as n (%)/n (%); ^†^ Student’s *t*-test for quantitative unpaired data; chi-square tests for categorical data. ^§^ Normalization of ferritin using “Ferritin level/dosage age ratio,” as described by [27].

## Data Availability

Data sharing not applicable.

## Supplementary Materials

The following supporting information can be downloaded at: https://www.mdpi.com/article/10.3390/ijms23031304/s1.
