# Supplementary material for: SEC23B Loss-of-Function Suppresses Hepcidin Expression by Impairing Glycosylation Pathway in Human Hepatic Cells
Source: Int J Mol Sci. 2022 Jan 24;23(3):1304. doi: 10.3390/ijms23031304 (PMC8835815; doi:10.3390/ijms23031304)
Supplement: Supplementary file 1 [file ijms-23-01304-s001.zip › ijms-1554361-supplementary.pdf]

SEC23B loss-of-function suppresses hepcidin expression by affecting glycosylation pathway in hepatic cells

Barbara Eleni Rosato [1,2], Roberta Marra [1,2], Vanessa D'Onofrio [1,2], Federica Del Giudice [2], Simone Della Monica [1,2], Achille Iolascon [1,2], Immacolata Andolfo [1,2,†*] and Roberta Russo [1,2,†*]

[1] Dipartimento di Medicina Molecolare e Biotecnologie Mediche, Università degli Studi di Napoli Federico II, 80131, Napoli, Italy; rosato@ceinge.unina.it (B.E.R.); robertamarra.r@gmail.com (R.M.); vanessa.donofrio2@gmail.com (V.D.); simonedellamonica28@gmail.com (S.D.M.); achille.iolascon@unina.it (A.I.)

[2] CEINGE Biotecnologie Avanzate, 80145, Napoli, Italy; rosato@ceinge.unina.it (B.E.R.); robertamarra.r@gmail.com (R.M.); vanessa.donofrio2@gmail.com (V.D.); delgiudicef@ceinge.unina.it (F.D.G.); simonedellamonica28@gmail.com (S.D.M.); achille.iolascon@unina.it (A.I.); immacolata.andolfo@unina.it (I.A.); roberta.russo@unina.it (R.R.).

* Correspondence: roberta.russo@unina.it (R.R.); immacolata.andolfo@unina.it (I.A.)

† These authors equally contributed

Supplemental data contains:

– Figure S1

– Figure S2

– Supplemental material and methods

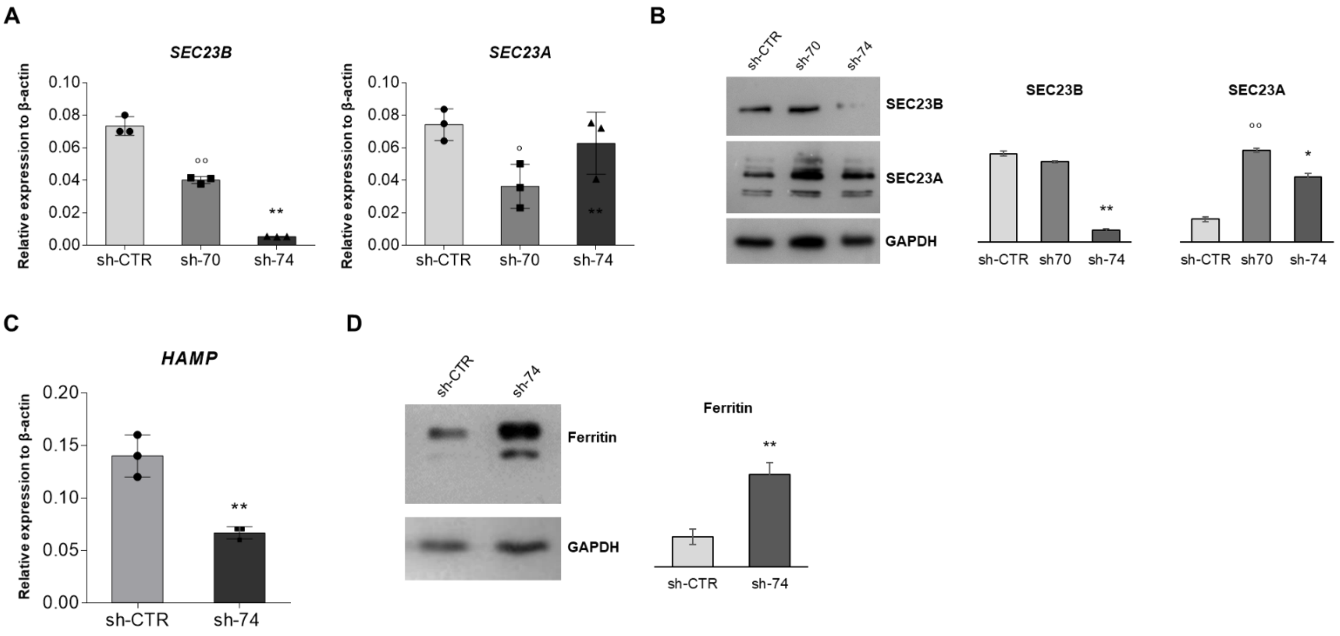

Figure S1. Characterization of HepG2 silenced for SEC23B. (A) *SEC23B* and *SEC23A* genes expression in HepG2 clones silenced for *SEC23B*, i.e., sh-*SEC23B*-70 (sh-70) and sh-*SEC23B*-74 (sh-74), compared to control cells (sh-CTR). Data are means ± standard deviation of three independent experiments (**p < 0.01, sh-CTR vs sh-74; °°p < 0.01, sh-CTR vs sh-70; °p < 0.05, sh-CTR vs sh-70; Student's t test). **(B)** Representatives immunoblot of SEC23B and SEC23A in HepG2 sh-70, sh-74, and sh-CTR cells (left panel) and densitometry quantification (right panel). Data are means ± standard deviation of three independent experiments (*p < 0.05, sh-CTR vs sh-74; **p < 0.01, sh-CTR vs sh-74; °°p<0.01, sh-CTR vs sh-70; Student's t test). **(C)** *HAMP*, gene expression in HepG2 sh-74 compared to HepG2 sh-CTR cells. Data are means ± standard deviation of three independent experiments (**p < 0.01; Student's t test). **(D)** Representatives immunoblot of Ferritin in HepG2 sh-74 compared to HepG2 sh-CTR cells, and densitometry quantification. Data are means ± standard deviation of three independent experiments (**p < 0.01; Student's t test).

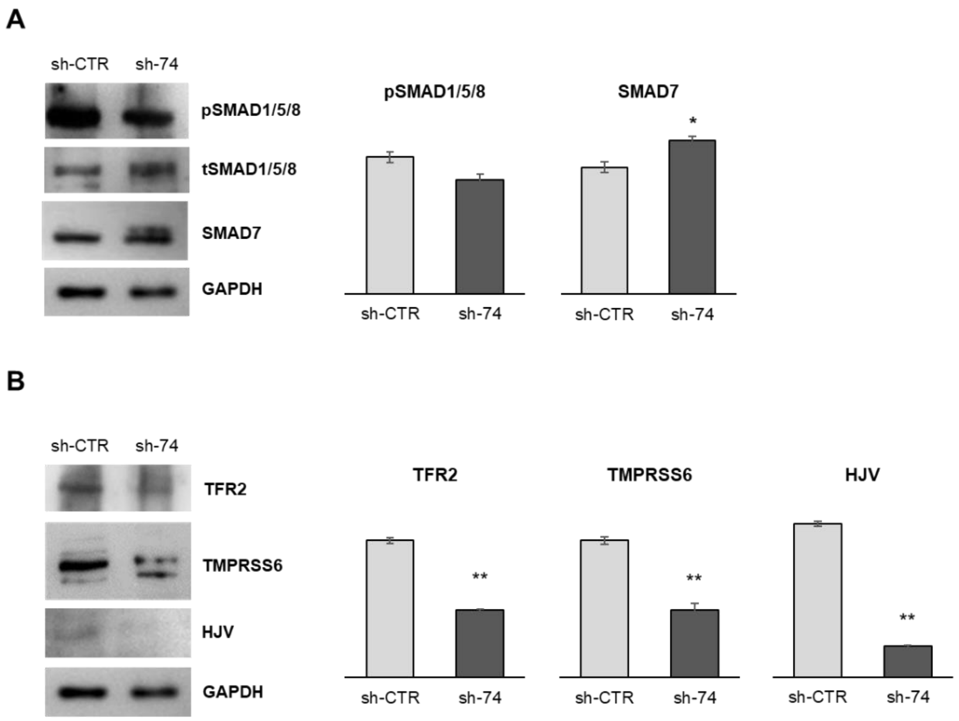

Figure S2. **Evaluation of the activation of BMP/SMADs pathway in SEC23B-silenced HepG2 and membrane proteins expression.** (A) Left panel. Representative immunoblots of pSMAD1/5/8, tSMAD1/5/8 and SMAD7 proteins in total cells lysate of HepG2 sh-74 and sh-CTR cells. GAPDH is the loading control. Right panel. Quantification by densitometric analysis of three separate Western blots with similar results. Data are means ± standard deviation (*$p < 0.05$; Student's t test). (B) Left panel. Representative immunoblots of TFR2, HJV, and TMPRSS6 proteins in total cells lysate of HepG2 sh-74 and sh-CTR cells. GAPDH is the loading control. Right panel. Quantification by densitometric analysis of three separate Western blots with similar results. Data are means ± standard deviation. (**$p < 0.01$; Student's t test).

Supplemental Material and Methods

HepG2 cells were obtained from American Type Culture Collection (ATCC, Manassas, VA, United States) and maintained following the manufacturer's instruction. [1,2] The wild-type (control) HepG2 cells and *SEC23B*-silenced HepG2 stable clones were cultured in RPMI 1640 (Sigma Aldrich, Milan, Italy) supplemented with 10% (v/v) fetal bovine serum (Life Technologies; California, USA), 100 U/mL penicillin (Life Technologies), and 100 mg/mL streptomycin (Life Technologies), at 37°C in humidified air/CO2 (19:1) atmosphere.

References

1. Russo, R.; Marra, R.; Andolfo, I.; De Rosa, G.; Rosato, B.E.; Manna, F.; Gambale, A.; Raia, M.; Unal, S.; Barella, S.; et al. Characterization of Two Cases of Congenital Dyserythropoietic Anemia Type I Shed Light on the Uncharacterized C15orf41 Protein. *Front Physiol* **2019**, *10*, 621, doi:10.3389/fphys.2019.00621.
2. Andolfo, I.; Rosato, B.E.; Manna, F.; De Rosa, G.; Marra, R.; Gambale, A.; Girelli, D.; Russo, R.; Iolascon, A. Gain-of-function mutations in PIEZO1 directly impair hepatic iron metabolism via the inhibition of the BMP/SMADs pathway. *Am J Hematol* **2020**, *95*, 188-197, doi:10.1002/ajh.25683.
